# The Song of the Peripatetic

**Published:** 1896-11

**Authors:** F. E. Daniel


### THE SONG OF THE PERIPATETIC.

AIR: MARCH TO MOSCOW. (Home made.)

Oh, I'm a surgeon bold and a doctor old,
Of the physio-school eclectic,
Where they teach in a week how to cure the sick,
The lame, the halt, the afflicted.
  No diploma have I, nor certificate; why
Should I? I've cheek,—that's sufficient:
And of lecture halls and college walls

My knowledge, too, is deficient.
What care I for them? or they for me?
So I boast and blow and deceive?
To shut the eye and open the purse,—
That's my aim and my game. I believe
There are fools and dupes—and wise ones too,
Who'll come to my net by the score,
If I only look wise,—talk big—advertise
In a way never thought of before.
Mississippi, Alabama and old Illinois
Are too wise to be gulled by my racket;
Tho' many a year without hinder or fear
I've practiced there,—fllling my pocket.
Laws they have made, in those States, it is said,
In town and country and city,
Which for the "Pratice's" sake surely do take the cake;
I had to get-up-and-get; what a pity!
So, to Texas I'll hie me; there too I shall buy me
A license,—and duly record it;
With plenty to do there's nobody who
Than I, can better afford it.
The climate is mild and the laws too—(the fact is—)
Are loose, and don't hinder the practice.
And nobody cares and nobody knows
Whither I *comes*—or whither I *goes*,
But everyone minds his own bizness!
So, I'll hie me away to this country so gay,—
The land of pe–rairie and plenty:
For I'm a surgeon bold and a doctor old,
(Though my learning 's a little scanty),
And to "bleed" the sick and "cripple" the lame
By my ways that are dark and uncanny,—
Shall be my aim; in the end tis the same,
And I'll bully the doctors in Texas.
Though no diploma have I, nor certificate; why
Should I? It don't matter a penny.
Austin, they say, is lively and gay,—
The people are "flush" and familiar;—
And no "pathy" is there that can compare
With our own old glorious "similiar;"
There, printing is cheap: I've plenty of cheek
Adamantine and awful flinty;

So with plenty of cash I'll cut a swell dash,
And capture the town in a jiffy.
I''ll take in the simple and humbug the wise—
With my oily tongue and ready-made lies.
Cancer I'll cure; and consumption sure
I'll make them believe I can master.
Those horrid old tumors and ugly blood humors
I'll doctor, I'll cut and I'll plaster.
Then I'll away to the West—quite safe from arrest
With my pockets all full of their ducats,
Before they know that cancer will grow
Right back on the spot whence I took it.
Let them bleed to death; I don't care—a breath,
Or cough up their lungs—or their liver,
That mother's forlorn for her baby still-born
On account of the stuff that I gave her.—
So ignorance is bliss and cheek is success,—
I'll hie me away to the prairie:
Austin's the Queen, the fairest e'er seen,
There money is plenty and people are green,—
No laws to oppress nor rob me of rest
I'll practice my wiles like a daisy,
Till the last bottom dollar is safe in the hollow
At the bottom of pockets so deep.
True, my victims will sigh;—the duped ones will cry;
O'er the fruits of their folly they'll weep;
And wher'er I'm trusted *somebody'l be busted*,—
If they make it too warm for my stay,
I'll away to the West far away from arrest,
And let them console as they may.
For,—I'm a surgeon bold and a doctor old,
With plenty of cheek, and assurance sleek—
Too wise to be duped, too old to be caught—
There being no laws, why should I fear ought?—
So, I'll practice a while in Texas.

F. E. Daniel.

